# Evolutionary Restructuring and Systematic Review of the *NBPF Gene Family*: Comparative Genomics, Functional Divergence, and Disease-Linked Pathways

**DOI:** 10.3390/jdb14010010

**Published:** 2026-02-24

**Authors:** Manuel Escalona, Rosa Roy

**Affiliations:** Departamento de Biología, Universidad Autónoma de Madrid, 28049 Madrid, Spain; manuel.escalona@estudiante.uam.es

**Keywords:** *NBPF1*, *NBPF26*, *NBPF14*, evolution, neoplasms, congenital diseases, bone growth disorders, neurological disorders

## Abstract

The Neuroblastoma Breakpoint Family (NBPF) consists of 23 genes, 9 of which are pseudogenes, and is characterized by extensive duplication events and species-specific diversification in *Homo sapiens*, as well as by the presence of a unique protein domain known as Olduvai (also referred to as DUF1220 or the NBPF domain). Previous studies have attempted to define subfamilies based on the presence of HLS triplet domains; however, this classification has become increasingly unclear with the identification of additional *NBPF* members. The family remains poorly understood, and the functions of many genes are still unknown, although several have been hypothesized to play key roles in cell proliferation and developmental processes, particularly in neural and skeletal tissues. In this study, we systematically analyzed all available data on the *NBPF* gene family using the PRISMA-S methodology to infer the biological functions in which these genes may be involved. We also generated multiple phylogenetic trees to support the creation of coherent subfamilies and to correlate the origin of each subfamily with homologous genes in our last common ancestor with the *Pan* genus, providing what we believe to be one of the most comprehensive phylogenetic reconstructions including all currently annotated NBPF members. Through comparative genomic and phylogenetic analyses, we propose that the *NBPF* may have originated from a duplication of the *PDE4DIP* gene, with *NBPF26* representing the ancestral member from which the remaining NBPF genes diverged via lineage-specific segmental duplications. In this systematic review and comparative genomic study, we present the first integrative synthesis of our knowledge of the *NBPF*, encompassing its evolutionary origins, structural dynamics, expression across tissues, and clinical associations.

## 1. Introduction

The Neuroblastoma Breakpoint Family (NBPF) genes constitute a group whose functions and signalling pathways remain largely unknown. They have been proposed as candidate genes involved in the pathological development of neurological, oncogenic, and skeletal disorders, as well as rare conditions such as Brugada syndrome and pituitary stalk interruption syndrome (PSIS) [1,2,3].

**Figure 1:**
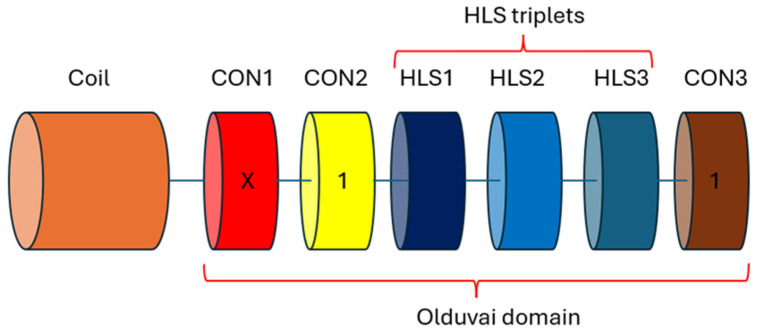
Structure of an NBPF protein, showing an N-terminal coiled-coil region followed by a variable number of Olduvai domains composed of multiple CON1 subdomains, one CON2 subdomain, several HLS triplets (HLS1–HLS2–HLS3), and one CON3 subdomain.

A defining feature of NBPF genes is the presence of a unique domain known as Olduvai (also referred to as NBPF or DUF1220). This domain comprises approximately 65 amino acids, and its biological function has not yet been determined [4]. Based on sequence similarity, Olduvai domains are classified into two main groups: CON (CON1, CON2, and CON3) and HLS (HLS1, HLS2, and HLS3) [5]. These correspond to conserved sequences shared with other primates (CON) and human lineage-specific sequences (HLS). *NBPF* genes typically contain an N-terminal coiled-coil region followed by a variable number of Olduvai domains. Each Olduvai domain contains a variable number of CON1 subdomains, one CON2 subdomain, a variable number of HLS triplets (HLS1–HLS2–HLS3), and one CON3 subdomain (Figure 1).

The Olduvai domain has been associated with neurological development in a dosage-dependent manner, with increased copy number variation (CNV) linked to a higher risk and severity of autism and schizophrenia [4,5,6]. However, the current evidence is not sufficient to establish a conclusive relationship between NBPF genes and these complex, multifactorial diseases.

Several studies have shown that the enzyme furin can cleave CON1 subdomains and HLS triplets in NBPF pro-proteins. These findings suggest that disorders such as autism may be influenced by the total number of Olduvai copies in the genome, rather than variation in a single NBPF gene. They also support the possibility of coregulation between NBPF genes and NOTCH2NL genes [7,8].

In this study, we reviewed the literature on the evolution of NBPF members and analyzed their sequences at multiple levels (including cDNA, coding sequences, proteins, and genomic DNA) to generate new phylogenetic trees that clarify their evolutionary history. We used the myomegalin gene (*PDE4DIP*) as an outgroup and as a potential ancestral gene from which the NBPF family may have originated, due to their historical association, partial sequence similarity, and the presence of a single Olduvai copy in *PDE4DIP* [5]. In addition to *PDE4DIP*, we included the fusion genes *NOTCH2NLR* + *NBPF26*, *NOTCH2NLB* + *NBPF14*, *NOTCH2NLA* + *NBPF10*, and *NOTCH2NLC* + *NBPF19*, as they are extremely similar to *PDE4DIP*. Previous studies have proposed that the NBPF family coevolved with NOTCH2NL genes because these genes originated from altered segmental duplications of *PDE4DIP* [9]. To our knowledge, this is the first study to include all currently known NBPF members and their associated NOTCH2NL genes in a phylogenetic analysis.

We also aimed to compile all the available information on the pathological roles of NBPF genes, as the family was originally named after a characteristic mutation of *NBPF1* detected in neuroblastoma.

*NBPF1* is the most extensively studied member of the family, partly due to a characteristic translocation found in neuroblastoma patients. It has recently been shown to interact with Chibby, exerting a negative regulatory effect on the Wnt signalling pathway [10]. The promoter region of *NBPF1* is responsive to the transcription factor NF-κB, and the protein is primarily located in the cytoplasm [10,11]. This suggests that *NBPF1* could function as a transcription factor capable of translocating to the nucleus upon activation, although further studies are needed to confirm this hypothesis.

## 2. Materials and Methods

### 2.1. Sequence Analysis and Alignment

For this study we extracted the cDNA, CDS and genomic sequence data of all the analyzed genes from the latest build of NCBI’s Ensembl (Table 1) (current release 113—October 2024) [12], and we extracted the amino acid sequences from Uniprot’s database [13].

The reasoning behind the wide selection of analyses performed relates to the need to obtain as much information as possible for the development of a more comprehensive analysis. Genomic DNA analysis allows for a better understanding of how different gene sequences are conserved in this family, making it easier to identify possible gene duplications and the origins of each NBPF gene. The analysis of CDS and cDNA sequences was necessary for a functional and structural approach. CDS sequences analysis allows for the direct comparison of the coding sequence of each gene, grouping the more similar sequences together. Meanwhile, cDNA analyses are useful because they show how similar the sequences analyzed are beyond the nucleotides that are going to be traduced into an aminoacidic sequence, allowing for the comparison of 3′UTRs (un-translated region) between NBPF genes. Last but not least, the analysis of amino acid sequences is crucial for a functional grouping of these genes and even supports other analyses because of the presence of multiple promoter regions in some *NBPF* genes.

To align the different sequences, we used the multiple alignment programme for amino acid or nucleotide sequences, MAFFT version 7 [14,15], with the following settings: uppercase/lowercase was set to the “same as input” and the direction of nucleotide sequences was set to “adjust direction according to the first sequence (accurate enough for most cases)”. The advanced settings were set to “leave gappy regions” in the align unrelated segments, too? The rest of the settings were left at default values.

After performing the genomic alignments, we used MAFFT version 7 [14,15] to generate two trees with different methods for each alignment: one with neighbour joining because it is compatible with bootstrap settings (which we set to “1000” in the number of resampling section), and one with UPGMA to include the pseudogene *NBPF2P*. This pseudogene is the only member of its family that does not share a conserved region with the other members, meaning that it cannot be analyzed via neighbour joining strategies. In this study we propose an inferred position of *NBPF2P* within the phylogenetic tree, but further studies are required to correctly assess its relationship with the other members of its family. The bootstrap thresholds used were >70 for strong support, 50–70 for moderate support and <50 for weak support.

We chose neighbour joining methods over other methodologies because they calculate distances between sequences instead of rooting them to a single ancestral sequence and have better bootstrap analysis support than most of the other methods, with statistically stronger results than other methodologies. The reasoning behind choosing UPGMA methods over other kinds of analysis is that this methodology recalculates the distances between nodes after each iteration, which makes less likely to get stuck in a local maximum than other methodologies like Bayesian methods.

To analyze the CDS, cDNA and protein alignments, we used UPGMA methods (incompatible with bootstrap analysis) due to the variance within transcripts and the presence of multiple promoter regions in some members, which leave no gap-free sites for neighbour joining methods. A Jukes–Cantor substitution model was used for the genomic DNA, CDS and cDNA trees. This substitution method uses the same substitution rate for each nucleotide and each position, making it a good option due to the closeness of the NBPF genes and the relatively short time in which the gene family has changed and diverged. Meanwhile, a JTT substitution method was used for the generation of the protein tree. The JTT substitution method is based on a comparison of observed proportions of amino acid pairs between pairs of sequences, calculating the divergence between these sequences via a maximum likelihood method.

We used the sequences shown in Table 1, which include the specific gene, the Ensembl code, the genomic location (*Homo sapiens* and *Pan troglodytes*) and the corresponding chromosomal band. The *Pan troglodytes* genes used were selected via orthologue sequences for each human NBPF as suggested by the Ensembl database.

### 2.2. Search Methods and Flow of Article Review

For each gene, we extracted all phenotype-related data from Ensembl (current release 113—October 2024) [12] and Genecards [16]. In Ensembl we searched each gene, exported cDNA, CDS and genomic DNA sequences and checked for phenotypes in the section “Ontologies: Phenotypes”. In Genecards we extracted a summary of each gene functions and its associated disorders under the section “Disorders”. After collecting all the data, we used the PRISMA-S method to search for publications that described pathologies related to the NBPF members using the PubMed, ScienceDirect and Google Scholar databases. For PubMed and ScienceDirect we used a search string consisting of inputting the name of each specific gene, e.g., “*NBPF1*”, “*NBPF3*”, and “*NBPF4*”. After the first selection of articles, we included two more search strings to obtain additional historical data of the family, and the structural and molecular data of the NBPF domain, as follows: “NBPF” and “NBPF domain”. In the filter section of PubMed and ScienceDirect we chose to not apply any filters and to use all published articles independently from the year of publication.

In this study we decided to use Google Scholar as an assistant database as we had low expectations with regard to finding enough articles for each *NBPF* gene. We used the exact same search strings as for PubMed and ScienceDirect. The only difference with the other curated databases was that we had to verify that the articles found came from peer-reviewing publishers. We finally excluded all articles that were not published in English for an easier approach using this tool.

For this study, any study registries were consulted, and citation searching or contacting other authors was undertaken.

The update method involved re-searching on the same platforms, and the last search was made on 20 September 2025.

**Figure 2:**
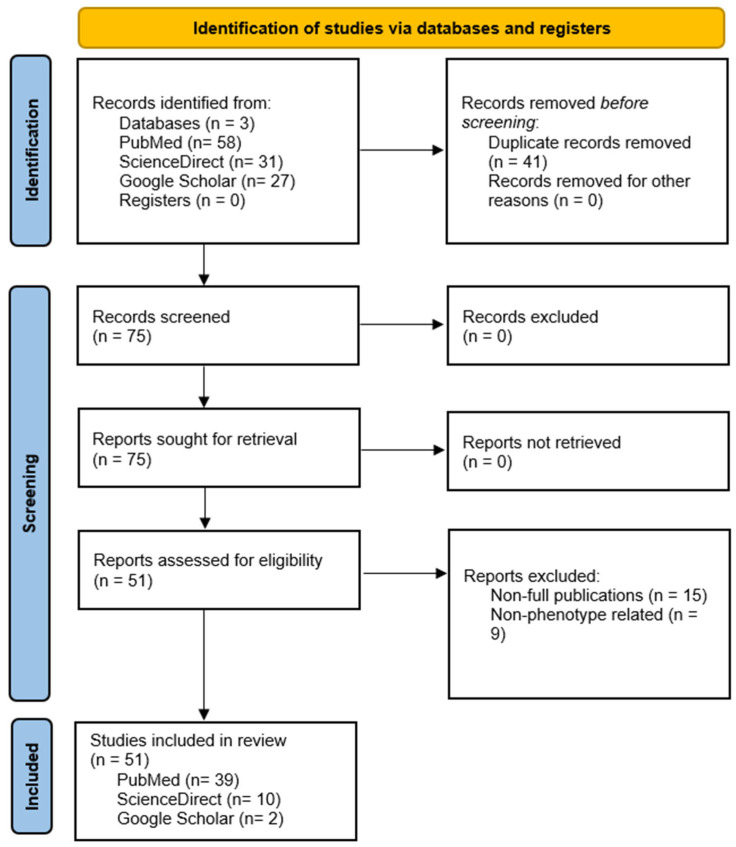
Prisma workflow for the validation and final acceptance of articles for this review.

After the initial data collection, we excluded any repeated articles retrieved from multiple platforms by manually removing the duplicated articles. Then we excluded non-complete articles, which were result of the search strings used and included entries that contained only the abstract or early results of non-published articles. Finally, we excluded articles in which there was not a direct or theoretical correlation with the phenotype of *NBPF* genes. In the end, we used a total of 51 articles for the pathological processes involving the *NBPF*. This process is explained in Figure 2.

All articles used for this study are visible in Table S1 (Supplementary Materials). Both the authors of this study have reviewed all the articles selected.

## 3. Results

To comprehensively depict the evolutionary and biomedical dimensions of the NBPF gene family, we present our results through two main approaches. First, we constructed phylogenetic trees based on genomic DNA, cDNA, CDS, and protein sequences to elucidate evolutionary relationships among the 23 NBPF members. Second, we examined the potential involvement of each NBPF gene in various human diseases, including neurological disorders, multiple oncological processes, skeletal abnormalities, and other rare conditions.

### 3.1. Phylogenetic Associations Between Members

#### 3.1.1. Genomic DNA Trees

**Figure 3:**
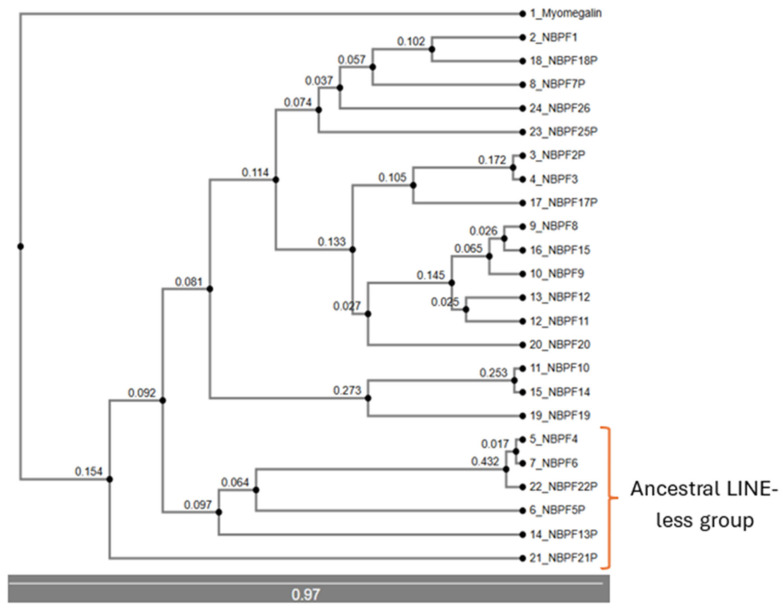
Genomic DNA phylogenetic tree obtained through UPGMA methods (not compatible with bootstrap analysis) showing the evolutionary distance of each node from the previous one and a scale.

Rooting with myomegalin (*PDE4DIP*) (Figure 3), and using UPGMA methods, we can distinguish the first group as consisting of *NBPF21P*, *NBPF13P*, *NBPF5P*, *NBPF22P*, *NBPF6*, and *NBPF4*. The *NBPF6* and *NBPF4* genes are characterized by the absence of a long interspersed nuclear element (LINE) in their second intron, which is present in the rest of the members of this family [9]. The next division appears as two branches: one containing *NBPF1*, *NBPF18P*, *NBPF26*, *NBPF25P*, *NBPF7P*, *NBPF10*, *NBPF14* and *NBPF19*, and another including *NBPF2P*, *NBPF3*, *NBPF17P*, *NBPF8*, *NBPF9*, *NBPF15*, *NBPF11*, *NBPF12*, and *NBPF20*.

**Figure 4:**
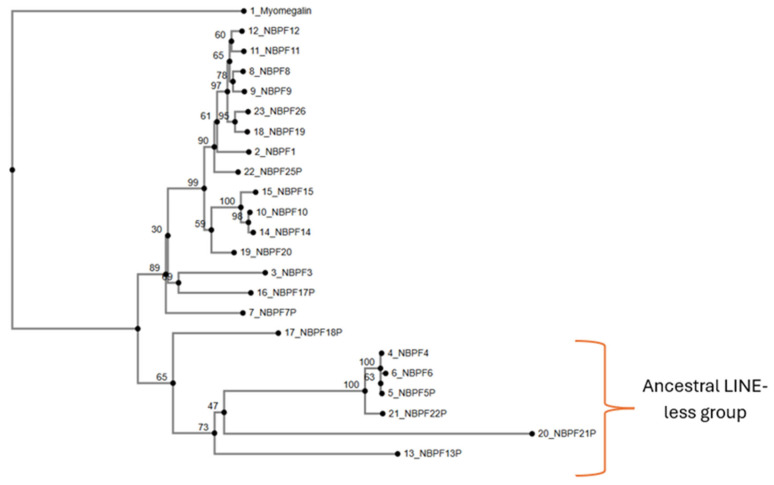
Genomic DNA phylogenetic tree obtained through neighbour joining methods with its bootstrap results for each branch (*NBPF2P* was excluded because alignment has no gap-free spaces, which are needed for the neighbour joining methods). (This figure was modified for quality reasons; the original is available in the Supplementary Materials, Figure S1).

When neighbour joining methods are used, we observe differences in cluster organization (Figure 4). The group composed of *NBPF21P*, *NBPF13P*, *NBPF5P*, *NBPF22P*, *NBPF6*, and *NBPF4* is similar to the aggrupation obtained through UPGMA methods. *NBPF5P* is associated with *NBPF6*, but its bootstrap score is only 53 (indicating moderate-to-weak support). The rest of this group, marked as an ancestral LINE-less group, shows a clustering similar to the UPGMA tree (Figure 3). *NBPF18P* is closely associated with this LINE-less group, whereas in the UPGMA tree appeared closer to *NBPF1*. The other members show different cluster arrangements: on one hand, they appear with new associations resulting in a mix of the two well-defined branches of the UPGMA tree, and, on the other hand, some associations remain the same, like *NBPF11* and *NBPF12* (bootstrap score of 60), *NBPF10* and *NBPF14* (bootstrap score of 98), and *NBPF3* and *NBPF17P* (bootstrap score of 69).

#### 3.1.2. Genomic DNA + NOTCH2NL Tree

**Figure 5:**
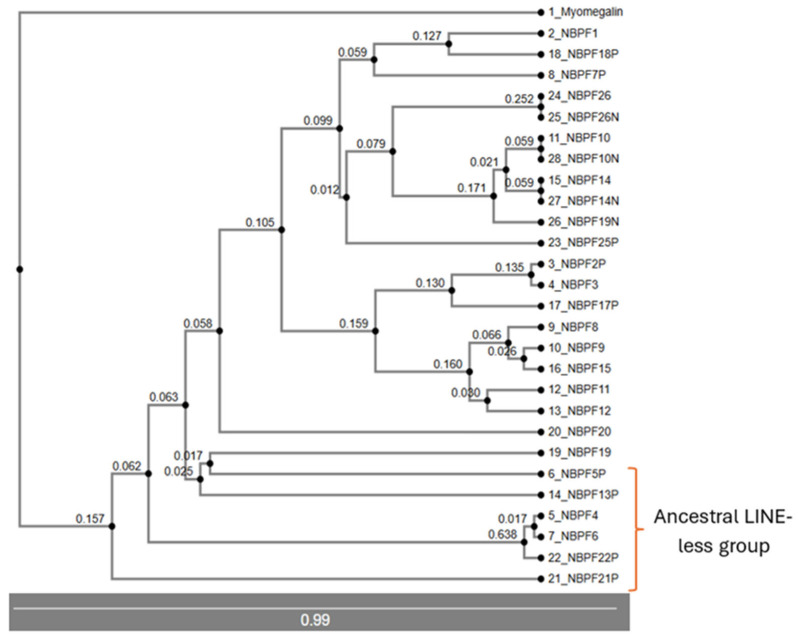
Genomic DNA + *NOTCH2NL* genomic DNA phylogenetic tree obtained through UPGMA methods (not compatible with bootstrap analysis) showing the evolutionary distance of each node from the previous one and a scale.

The genes *NBPF26*, *NBPF14*, *NBPF10* and *NBPF19* have an associated *NOTCH2NL* gene. When we added the sequences of the *NOTCH2NL* genes to the corresponding *NBPF* genes and rooted with myomegalin (*PDE4DIP*) (Figure 5), the resultant tree was different from the original and was the first phylogenetic reconstruction, to our knowledge, to include both *NBPF* + *NOTCH2NL* and only the *NBPF* segments of *NBPF + NOTCH2NL* genes. It was also the first phylogenetic tree including all members of the family to date.

In the UPGMA tree, the group composed of *NBPF21P*, *NBPF13P*, *NBPF5P*, and *NBPF22P*, *NBPF6* and *NBPF4* remain associated, which represent the most ancestral genes of this family.

Other important associations in this tree include *NBPF10*, *NBPF10N* (*NBPF10* and *NOTCH2NLA*), *NBPF14*, *NBPF14N* (*NBPF14* and *NOTCH2NLB*), *NBPF19N* (*NBPF19* and *NOTCH2NLC*), *NBPF26* and *NBPF26N* (*NBPF26* and *NOTCH2NLR*), *NBPF2P*, *NBPF3* and *NBPF17P* (consistent with Figure 3 and Figure 4), and *NBPF11* and *NBPF12* (consistent with Figure 3 and Figure 4).

**Figure 6:**
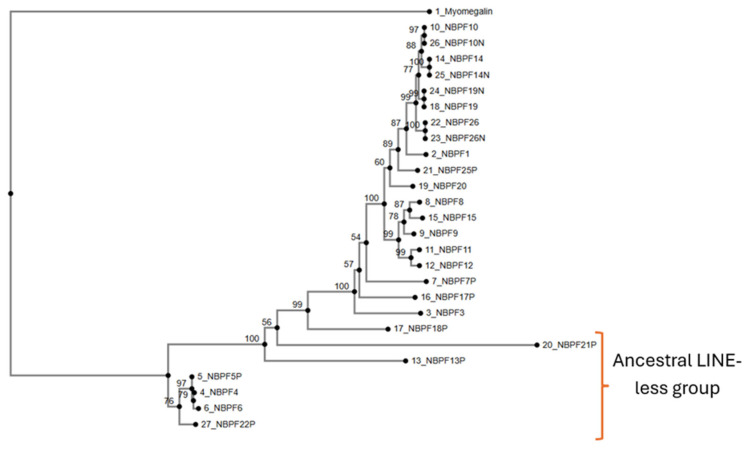
Genomic DNA + *NOTCH2NL* genomic DNA phylogenetic tree obtained through neighbour joining methods with its bootstrap results for each branch (*NBPF2P* was excluded because alignment has no gap-free spaces, which are needed for the neighbour joining methods). (This figure was edited for quality reasons; the original is available in the Supplementary Materials, Figure S2).

After applying neighbour joining methods (Figure 6), we observe certain differences. *NBPF19* is now grouped with *NBPF19N* (*NBPF19* and *NOTCH2NLC*) and within the group of *NBPF* genes that have an associated *NOTCH2LN* gene (*NBPF26*, *NBPF19*, *NBPF10* and *NBPF14*). This group has high bootstrap scores due to the inclusion of repetitive sequences (*NBPF* genes and *NBPFN* sequences), but the association between different genes is strongly supported, with bootstrap values ranging from 77 to 99. *NBPF1* and *NBPF25P* are closely related to this gene group with bootstrap values of 87 and 89, respectively. Two groups worth mentioning are the group composed of *NBPF21P*, *NBPF13P*, *NBPF22P*, *NBPF5P*, *NBPF4* and *NBPF6* (ancestral LINE-less group), and the group composed of *NBPF11*, *NBPF12*, *NBPF8*, *NBPF9* and *NBPF15*, as they are conserved across the different study methods (UPGMA and neighbour joining) as shown in Figure 3, Figure 5 and Figure 6.

#### 3.1.3. cDNA and CDS Trees

The NBPF family is known for its sequence conservation, making the phylogenetic trees obtained from their cDNAs and CDSs especially difficult to interpret. In these trees we included the known *NBPF* sequences of *Pan troglodytes* to explore a possible new conformation of the *NBPF* evolution. It is also worth noticing (Figures S3 and S4) that due to their spatial separation, some members have experienced multiple mutations and others are practically unchanged. For example, the gene *NBPF26* had transcripts that appeared in multiple branches of the resultant trees, reinforcing the idea that the original gene’s regulatory and coding sequences were similar to *NBPF26*.

From these phylogenetic trees we can also infer that the group composed of *NBPF6* and *NBPF4* had a common ancestor similar to *NBPF4* due to the association of this gene’s cDNA and CDS with ENSPTRG00000001028’s cDNA and CDS (*Pan troglodytes*); we rooted both cDNA and CDS trees using this group as root because they do not present a LINE sequence in their second intron and are considered the most primitive members of the family. Since the *NBPF3* transcripts are all associated with one another, they have a high correlation with the transcripts of *NBPF3* of *Pan troglodytes*.

#### 3.1.4. Protein Tree

**Figure 7:**
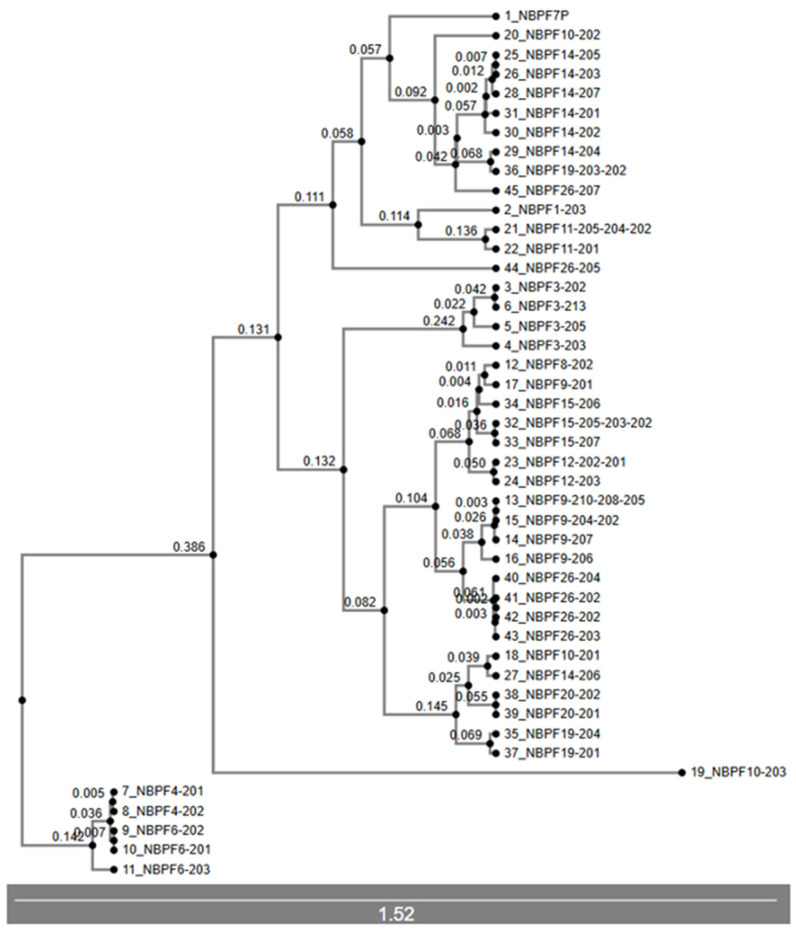
Protein phylogenetic tree obtained through UPGMA methods (not compatible with bootstrap analysis) showing the evolutionary distance of each node from the previous one and a scale.

The organization of this tree’s branches (Figure 7) is notable, and it has branches with the same disposition and others that are completely different from the ones obtained in the cDNA and CDS trees. As previously mentioned, this gene family shows extreme conservation between members, making synonymous mutations quite common, especially among the members within the 1q21 region. *NBPF26 NBPF10*, *NBPF14* and *NBPF19* appear dispersed across the tree’s branches. For the first time, *NBPF11* and *NBPF12* do not appear associated, generating two groups, one composed of *NBPF8*, *NBPF9*, *NBPF15*, *NBPF12*, *NBPF26*, *NBPF 10*, *NBPF14*, *NBPF20*, *NBPF19* and *NBPF3*, and another group composed of *NBPF7P*, *NBPF10*, *NBPF14*, *NBPF19*, *NBPF26*, *NBPF1*, *NBPF11* and *NBPF26*. This tree was rooted using the *NBPF6* and *NBPF4* groups because they do not present a LINE sequence in their second intron and are considered the most primitive members of the family.

Due to the great variance observed between phylogenetic trees, we will discuss the implications of each group and association generated in the Section 4 and Section 5, with this analysis being robust at the cluster level and requiring further studies to generate a fine-scale branching resolution.

### 3.2. NBPFs and Disease Associations

For an easier understanding of the synthesis of all the reviewed articles used in this study, this section is organized according to the following structure: specific gene data synthesis according to the section (i.e., Section 3.2.1. *NBPFs* associated with neurological pathologies in development) starting from *NBPF26* and continuing in descending succession. In Table 2 and Table 3, a strength of association section can be found to the simplify data collection of future studies of specific NBPF genes.

#### 3.2.1. NBPFs Associated with Neurological Pathologies in Development

The NBPF family has been associated with multiple processes during neurogenesis (Table 2). In the study of Davis et al. [4] with 215 multiplex autism individuals, they described a correlation between the copy number variants (CNVs) of the Olduvai domain (DUF1220) in the chromosome region 1q21 and the overall neuronal number, brain size, and brain mass. Cases were documented of patients with macrocephaly and autism presenting excess CNVs of the Olduvai domain. The mechanism behind this phenomenon remains unclear, but its authors hypothesize that extra Olduvai copies promote accelerated neurogenesis and neuronal overproduction, potentially leading to abnormal brain development. On the other hand, patients with microcephaly and schizophrenia showed reduced CNVs of the Olduvai domain, which has been linked to lower neuronal numbers and reduced cortical grey matter thickness [4]. These claims remain controversial, as they present CNVs of Olduvai subdomains as a primary explanatory factor for complex disorders such as autism and schizophrenia. Although supported by several references [5,6,17], further work is required to provide a mechanistic explanation for how Olduvai CNVs modulate neurodevelopmental pathways. Keeney et al. [17] proposed that *NBPF* gene dosage affects cell proliferation, particularly during neurogenesis, and may regulate mitochondrial activity, a crucial component of primate neurogenic timing.

The effect of the CNVs of the Olduvai domain in brain development does not depend on the total number of Olduvai copies alone but is specifically dose dependent on duplications or deletions of specific subdomains such as HLS1 and CON1 [4,5,6,17]. The CON subdomains (CON1, CON2 and CON3) are evolutionary conserved, whereas the HLS subdomains (HLS1, HLS2 and HLS3) are human specific and interact with *NOTCH*-mediated neurogenesis pathways. In a study of a population with autism, CNVs affecting the CON1 subdomain showed a negative correlation with the social diagnostic score (SDS) and communication diagnostic score (CDS) [4]. Multiplex autism cases exhibited strong linear correlations with CNVs of Olduvai subdomains, suggesting that the underlying mechanism may be inherited rather than sporadic.

*NBPF1*, one of the genes with the widest CNV range for the CON1 subdomain (estimated between 8.5 and 28.5 copies), is of particular interest due to its potential contribution to the dose-dependent regulation of neurodevelopment [4]. Keeney et al. [17] demonstrated that *NBPF1* overexpression strongly downregulates 52 proteins and upregulates 27, reducing mitochondrial activity. This reduction is hypothesized to contribute to the maintenance of neuron size, a hallmark of primate brains. According to this view, Olduvai domain dosage may act as a “molecular switch” as the authors claim, restricting cellular energy availability, standardizing neuronal size across populations, and promoting proliferation over hypertrophic growth. The findings of Keeney et al. [17] are still a preprint funded by the National Institute of Health (NIH), and remain to pass a peer review process. Nevertheless, their results are promising, and their conclusion is strongly supported by other published articles.

Other family members can have different effects on brain development. *NBPF14* shows sex-specific expression differences in the anterior cingulate cortex within the frontal cortex, a brain region with marked sex-biassed gene expression. This study proved that lithium treatment response in bipolar disorder could be predicted in women based on *NBPF14* expression [18], although further validation is required before considering *NBPF14* as a biomarker for female bipolar disorder.

*NBPF15*, also located in the 1q21 region, is a key candidate gene in a congenital disorder caused by the microduplication of this locus. The affected patient presented developmental delay, craniofacial dysmorphism, congenital heart disease, and sensorineural hearing loss [19].

*NBPF19* represents an unusual case. It has coevolved with *NOTCH2NLC* and possesses a transcript in which the *NOTCH2NLC* promoter drives a sequence that terminates with the *NBPF19* polyadenylation signal. Initially defined as a single fusion gene, these are now considered two separate genes. Ishiura et al. [20] showed that the deletion of a 5′UTR fragment of *NOTCH2NLC* is associated with neuronal intranuclear inclusion disease (NIID), although further studies are required to confirm whether *NBPF19* participates in this mechanism.

The studies of Arcos-Burgos et al. [21] and Alharbi et al. [22] proposed that single nucleotide polymorphisms can be linked to diseases like myalgic encephalomyelitis/chronic fatigue syndrome and autism, being rs3897177 (from *NBPF1*), rs1553120233 (from *NBPF10*) and rs200632836 (from *NBPF15*) polymorphisms with a strong association with the development of the diseases [21,22].

#### 3.2.2. NBPFs Associated with Oncological Processes

Multiple members of the NBPF family have been associated with different neoplasms (Table 2). Most of them have tissue-dependent dual roles, acting either as oncogenes or tumour suppressor genes depending on the cellular context.

Yao et al. [23] identified *NBPF26* as a “potential driver mutation gene” in hepatoid adenocarcinoma of the lung (HAL) in their study of whole-exome sequencing in one patient of non-alpha-fetoprotein-elevated HAL. In another study involving 271 patients of lung squamous cell carcinoma (LUSC), Wang et al. [24] classified *NBPF26* and six other genes as positive prognostic factors. *NBPF26* is also considered essential for the renewal and proliferation of radial glial cells during brain development, likely due to its proximity and co-expression with *NOTCH2NLR*.

Another gene that is affected in pulmonal neoplasms is *NBPF20*, with detectable mutations in 46.51% of the patients with multiple primary lung cancer (MPLC) and lymph node metastasis (LNM) [25]. *NBPF20* has also been identified as mutated in acute myeloid leukemia (AML), with alterations affecting its transcript leader sequence in 10.1% of patients studied [26].

Other family members appeared mutated in studies of different neoplasms. *NBPF15* is significantly downregulated in pancreatic ductal adenocarcinoma (PDAC) and, together with other classifier genes, can stratify patients into high-risk and low-risk groups, where the high-risk group shows higher tumour mutational burden (TMB) and increased tumour stemness scores [27]. *NBPF15* is also one of the seven genes associated with mixed ductal-endocrine carcinoma and neuroblastoma as inferred by the Malacards database [28]. *NBPF14* is among the most-frequently mutated and overexpressed genes in thymoma [29] and has specific mutations associated with the translocation of *NUP98* in AML [30]. Some of the NBPF members have only been described with altered expression patterns in specific oncological processes, due to the lack of information about their function and interaction with other proteins. This is the case for *NBPF9*, which is upregulated in all four stages of lung adenocarcinoma (ADC) [31], and *NBPF10*, which is upregulated in glioblastoma multiforme (GBM) [31], but downregulated in small-cell carcinoma (SCC) [32]. This gene has also been reported with mutations in 20% of the analyzed patients with hepatocellular carcinoma [33].

*NBPF12* has been studied in triple-negative breast cancer in a population in Nigeria and Barbados, revealing a high frequency of mutations in patients [34]. A similar study was recently performed in the US midwestern population, showing that *NBPF1* and *NBPF10* were commonly mutated and African American patients had a worse prognosis when these genes were altered [35].

*NBPF8* has been studied in non-Hodgkin B-cell lymphoma, and like most genes of that study, it had intragenic exon rearrangements (IERs), with exon 14 after exon 19 [36]. The lncRNA of *NBPF7* has shown effects over the cell cycle and tumorigenesis; in the study of Zhu et al. [37] an induced KO of a-catenin in the HaCaT cell line (keratinocytes) increased the proliferative capacity of those cells and the expression of *NBPF7*, due to the activation of the NF-kB pathway. When they induced a double KO for a-actin and *NBPF7* in the same cell line, the pro-proliferative effect was reverted, proving that *NBPF7* is a signal mediator of a-actin and proliferation in keratinocytes and that, by co-immunoprecipitation, *NBPF7* was colocalized inside the nucleus in association with P65 [37].

*NBPF1* is the most widely studied and frequently altered gene in the family, initially identified through a characteristic chromosomal translocation in a neuroblastoma patient, which led to the naming of the gene family. The main oncological process in which it has been studied is neuroblastoma and even with its pathways still being unclear, it has shown a high impact on the development of this kind of oncological process. In a recent study by Vandepoele et al. [38], it has been immunoprecipitated, making a complex with the proteins Chibby and clusterin; these proteins are part of a suppressive pathway of *Wnt* and form a tumour-suppressing complex. In a model of colorectal cancer (CRC), cells that overexpressed *NBPF1* had their proliferating capacities greatly reduced. This happened due to the inhibition of anchorage-independent growth as they were compared with a tumoral control group in which there was no inhibition of anchorage-independent growth [39]. In a neuroblastoma patient, the translocation t(1;17)(p36.2;q11.2) generated a fusion protein of the genes *NBPF1* and *ACCN1* (two antitumoral genes); this fusion protein lost all effect on inhibiting tumor development [39].

The deletion of the 1p36 chromosomal region, where *NBPF1* is located, is considered common in multiple kinds of neoplasms. Andries et al. [40] studied the effect of *NBPF1* overexpression and its aggregates in a wide range of oncological processes. The pathway comprehended the *NBPF1*-dependent cell cycle arrest in G1 and fate, which promotes the expression of *CDKN1A* (p21) mediated by p53. *NBPF1* is not expressed during mitosis. In neuroblastoma model cells, the effect of *NBPF1* overexpression in WT and p53KO cells was significantly different, in the WT group resulted in cell death in G1, and the p53KO group generated cell cycle arrest and *CDKN1A* induction independent of p53 [40]. In CRC model cells, the changes in protein expression were measured, showing 32 proteins (19 upregulated and 13 downregulated) with different concentrations. These proteins were associated with dermatological disease, inflammatory disease, inflammatory response, oncological processes, and endocrine system disorders, being 13 out of the most altered 14 genes associated with oncological processes. One of these proteins was S100P, which is related to drug resistance, metastasis, and poor clinical outcomes in prostate, breast and colon cancer [40,41]. In cervical cancer, the overexpression of *NBPF1* regulates the concentration of AKT1S1 and KIF1B (part of the PI3K/mTOR pathway), being commonly altered in ovarian cystic teratomas [42]. Methylations in the promoter region of *NBPF1* reduced its expression, showing the higher risk in the prognosis of patients with several types of oncological processes and especially with acute lymphoblastic leukemia [43,44].

The regulation of *NBPF1* is crucial to the prognosis of a patient in multiple oncological processes, as it has a protective function in most of them, like in CRC [41,45,46]. However, in other oncological processes it is a signal of a bad prognostic, such as in adrenocortical carcinoma (ACC) and lung adenocarcinoma (ADC) [32,45]. *NBPF1* downregulation is associated with methylations in its promoter region. The microenvironment of the tumours was studied in search of significant changes, showing mast cell infiltration in patients with lung adenocarcinoma (LUAD) and breast-invasive carcinoma (BRCA), improving the prognosis. However, CD8 T cells were significantly reduced in ACC patients with high *NBPF1* expression, explaining the worse prognosis of these patients [45]. In breast cancer, *NBPF1* appeared mutated whenever TP53 was already mutated, correlating with a higher metastatic risk [35].

#### 3.2.3. NBPFs Associated with Bone Growth Disorders

The NBPF family has been historically associated with issues in craniofacial bone and spine development, although the evidence remains limited due to the persistent lack of functional characterization of most proteins in the family. (Table 2). The principal genes associated with osteogenesis of this family are *NBPF1* and *NBPF15*. The microduplication of the 1q21 region that affects *NBPF15* has been reported to influence the development of craniofacial dysmorphism [19], and its overexpression is linked to mutations in its sequence, promoting osteogenesis in patients with orbital hypertelorism [47]. In *NBPF1*, there have been discovered mutations which overexpress the gene, promoting osteogenesis in patients with orbital hypertelorism, yet it is theorized to participate in more aberrant bone development processes [47]. Finally, *NBPF8* and *NBPF9* are candidate genes of mandibular development and the pathological development of macrognatism [48].

For a better global view of these groups of pathologies and the *NBPF* genes associated, Table 2 summarizes the Section 3.2.1, Section 3.2.2 and Section 3.2.3.

**Table 2:** Classification of pathologies in each group of study, the *NBPF* members associated with each one, references, type of evidence (genetic association (WES/exome/GWAS/CNV), functional/mechanistic, clinical observational (cohorts/signatures), case report, and review/database) and strength of association (strong (replicated + functional support), moderate (consistent multiple studies), and limited (isolated/preliminary)).

Group of Diseases	Specific Disease	*NBPF* Genes Implicated	References	Type of Evidence	Strength of Association
Neurological disorders	Macrocephaly, autism, microcephaly and schizophrenia	Olduvai domain copy number	[4,5,6,17,22]	Genetic association study (CNV in large cohorts); clinical observational study	Strong (linear associations replicated in multiple cohorts > 100 patients)
	Bipolarism	*NBPF14*	[18]	Gene expression predictive study (machine learning biomarkers)	Limited (lithium response prediction; no direct causality)
	Sensorineural hearing loss	*NBPF15*	[19]	Case report/index case (associated with 1q21 CNV)	Limited (isolated report in CNV context)
	Neuronal intranuclear inclusion disease	*NBPF19*	[20]	Genetic association study (repeat expansions in cohorts)	Moderate (finding in large NIID study)
Oncological development	HAL	*NBPF26*	[23]	Genetic association study (WES single case)	Limited (isolated hepatoid adenocarcinoma pulmonary case)
	LUSC	*NBPF26*	[24]	Clinical observational study (prognostic gene signature in IA/IB cohorts)	Moderate (part of validated signature in early LUSC)
	MPLC/LNM	*NBPF26*	[25]	Genetic association study (clonal trajectory in multiple primary lung cancer)	Limited (finding in specific clonal evolution)
	AML	*NBPF26* and *NBPF14*	[26,30]	Whole-exome sequencing/genetic association study (somatic deletions/rearrangements in pediatric cohorts)	Moderate (frequent in AML subgroups; poor prognosis)
	PDAC	*NBPF15*	[27]	Clinical observational study (risk model from single-cell + bulk sequencing)	Limited (part of predictive model; not main driver)
	Mixed Ductal-Endocrine Carcinoma	*NBPF15*	[28]	Review/database annotation (MalaCards)	Limited (database annotation; no primary study)
	Thymoma	*NBPF14*	[29]	Genetic association study (genetic characterization in thymomas)	Limited (finding in genomic analysis)
	ADC/GBM	*NBPF9* and *NBPF1/NBPF10*	[31,32]	Bioinformatics analysis (chromosomal alterations in lung cancers and GBM)	Limited (global chromosome 1 analysis; non-specific)
	SCC	*NBPF10*	[32]	Bioinformatics analysis (chromosome 1 genes in squamous lung carcinoma)	Limited (part of broad analysis)
	Hepatocellular carcinoma	*NBPF10*	[33]	Genetic association study (NGS in 39 Chinese HCC patients)	Limited (somatic mutations in small cohort)
	Triple-negative breast cancer	*NBPF12*	[34]	Genetic association study (genomic landscapes in Barbadian/Nigerian TNBC)	Limited (finding in ethnic genomic analysis)
	Breast cancer/BRCA	*NBPF10* and *NBPF1/NBPF1*	[35,45]	Clinical observational study (mutational landscape in breast cancer cohorts); pan-cancer analysis	Moderate (replicated across multiple cohorts and pan-cancer analyses)
	Non-Hodgkin B-cell lymphoma	*NBPF8*	[36]	Genetic association study (recurrent rearrangements in NHL)	Limited (finding in genetic study)
	CRC	*NBPF1*	[39,41,45,46]	Genetic association (translocation and pan-cancer signatures); Chinese mutational landscape	Moderate (multiple studies: functional + replicated genetics)
	Neuroblastoma	*NBPF1*	[39]	Genetic association study (constitutional translocation in NB patient)	Moderate (direct disruption in NB with functional support)
	ACC/LUAD	*NBPF1*	[45]	Pan-cancer analysis (oncogene/suppressor in adrenocortical and lung adenocarcinoma)	Limited (broad integrated analysis)
Bone growth disorders	Craniofacial dysmorphism	*NBPF15*	[19]	Case report/index case (1q21 CNV disorders)	Limited (isolated report)
	Orbital hypertelorism	*NBPF1* and *NBPF15*	[47]	Functional/mechanistic study (variant effects on osteogenesis)	Limited (preliminary Research Square study)
	Macrognatism	*NBPF8* and *NBPF9*	[48]	Clinical observational study (novel genes in Mediterranean families)	Moderate (familial linkage in mandibular prognathism)

#### 3.2.4. Other Diseases Associated with the NBPF Family (Table 3)

##### Diabetes

*NBPF20* has been associated with an increased susceptibility to type 2 diabetes mellitus (T2DM), promoting the apparition of the disease but never causing it. A key epigenetic marker linked to *NBPF20*’s role in T2DM is the hypomethylation of the CpG site cg26823705, observed in patients [49,50]. In women with polycystic ovary syndrome insulin resistance is higher than in the rest of the population, which may be related to *NBPF20* expression in skeletal muscle; this correlates with lower levels of c-peptide (part of pro-insulin) in these women.

Meanwhile, *NBPF1* has been investigated as a prognostic marker in type 1 diabetes mellitus (*T1DM*). The overexpression of *NBPF1* in peripheral blood mononuclear cells correlates with increased proportions of memory B cells, neutrophils, and CD4+ T cells, whereas eosinophil levels are negatively associated with *NBPF1* expression [51]. These immune cell distribution patterns suggest that *NBPF1* expression may influence T1DM prognosis by modulating the composition of circulating immune cells.

##### Brugada Syndrome

*NBPF11* and *NBPF12* have been implicated in Brugada syndrome, a disorder characterized by abnormal electrocardiogram patterns and an increased risk of sudden cardiac death in young adults. In patients with Brugada syndrome, the promoter regions of *NBPF11* and *NBPF12* exhibit insertions of human endogenous retrovirus K (HERV-K), with an approximately fivefold increase in HERV-K copy number compared to controls [1].

##### Pituitary Stalk Interruption Syndrome (PSIS)

Mutations in *NBPF10* and *NBPF9* were detected in the patients of PSIS. By whole-exome sequencing (WES), multiple mutations of *NBPF10* and *MUC4* were identified and shared between patients [2]. In *NBPF9*, the p.L279W variant is predicted to disable the gene’s protein function. In a subsequent cell culture, siRNAs targeting *NBPF9* to silence were added, significantly decreasing *LHX3* expression, which is an early transcription factor involved in the early pituitary differentiation [3].

##### Total Anomalous Pulmonary Venous Connection (TAPVC)

TAPVC is a congenital disease characterized by the non-connection of one or more pulmonary veins to the heart. During cardiac organogenesis, the gene *NBPF3* is upregulated and 7.7% of the patients studied show a duplication of this gene, making it one of the candidate genes involved in the pathological development of this disease [52].

##### Mayer–Rokitansky–Küster–Hauser Syndrome

This syndrome is characterized by vaginal agenesia due to abnormal development of the Müllerian ducts. The mutation (chr1: 145,291,369 G > A) has been detected in the promoter region of *NBPF10* and is associated with the syndrome; this region is also the 3′UTR region of *NOTCH2NLA*, a gene that has coevolved with *NBPF10* [53].

The summarized rare pathologies associated with some *NBPF* genes are shown in Table 3.

**Table 3:** Classification of other pathologies found during the study, the *NBPF* members associated with each one, references, type of evidence (genetic association (WES/exome/GWAS/CNV), functional/mechanistic, clinical observational (cohorts/signatures), case report, and review/database) and strength of association (strong (replicated + functional support), moderate (consistent multiple studies), and limited (isolated/preliminary)).

Group of Diseases	Specific Disease	*NBPF* Genes Implicated	References	Type of Evidence	Strength of Association
Metabolic disorders	Diabetes	*NBPF20*	[49,50]	Epigenetic association study (methylome-wide association in Korean cohorts); clinical observational study (DNA methylation changes in T2D/DKD)	Limited (differentially methylated sites associated with T2D risk in EWAS; no functional validation or replication across populations)
Cardiac disorders	Brugada syndrome	*NBPF11* and *NBPF12*	[1]	Genetic association study (metagenomic WGS analysis of viral integrations in Thai Brugada patients)	Limited (incidental finding in single-cohort WGS; unclear causal role vs. viral integration artefact)
Endocrine disorders	PSIS (Pituitary Stalk Interruption Syndrome)	*NBPF9* and *NBPF10*	[2,3]	Case report/index case (novel mutations in adults and whole-genome study of boy + family); clinical observational study	Limited (family-based reports suggesting involvement, small sample sizes, and preliminary evidence)
Congenital heart defects	TAPVC (Total Anomalous Pulmonary Vein Connection)	*NBPF3*	[52]	Genetic association study (rare CNV analysis in sporadic TAPVC cases)	Limited (identified in CNV screening; isolated cohort finding without replication)
Congenital malformations	Mayer-Rokitansky-Küster-Hauser syndrome	*NBPF10*	[53]	Genetic association study (de novo variants via WGS in MRKH patients)	Limited (de novo variants detected in small WGS cohort; no functional or replication data)

##### Limitations

Although the current knowledge of the *NBPF* remains limited, this review integrates the available evidence, much of which derives from clinical case studies involving complex diseases. Consequently, some of the interpretations proposed in the literature—and those discussed here—should be considered preliminary and subject to refinement as new data emerge. Recognizing these constraints, future research integrating comparative genomic analyses, functional studies, and multi-omics approaches will be crucial to validate the proposed hypotheses and clarify the biological functions and molecular pathways in which NBPF genes may be involved.

## 4. Discussion

The present review consolidates and extends current knowledge of the NBPF (Neuroblastoma Breakpoint Family) gene family, providing an evolutionary and biomedical framework for understanding one of the most enigmatic gene clusters in the human genome.

The phylogenetic origin of the NBPF genes is specially convoluted, being almost exclusive to mammals and some specific vertebrates like the Mexican tetra (*Astyanax mexicanus*). According to the Ensembl database, NBPF genes are present in some animals of the xenarthra superorder, disappearing in some species of the afrotherian superorder and carnivora order, including in hedgehogs, shrews and bats. The animal group with the greatest number of copies of NBPF genes is primates, with humans (*Homo sapiens*) being of special interest. In rodents, NBPF genes are almost entirely absent, except in some squirrels and marmots, whereas the rabbit (*Oryctolagus cuniculus*) exhibits nearly the same number of NBPF genes as humans, a peculiarity that warrants further investigation.

The analysis of phylogenetic trees enabled the formulation of a new hypothesis regarding the origin and evolution of the NBPF family. *PDE4DIP* is considered the original gene from which the NBPF family developed. One segment of the sequence of *PDE4DIP* was possibly copied in other regions, giving rise to new NBPF members such as *NBPF4*, *NBPF6*, *NBPF5P*, *NBPF21P*, *NBPF22P* and *NBPF13P*. This set of genes is highly conserved and consistently clustered together across analyses; for this, we have denominated them the “ancestral LINE-less group” due to the absence of a LINE insertion in their sequences [9]. Notably, this group contains all the NBPF members that are located outside of chromosome 1, suggesting that the retrotransposition of a *PDE4DIP* segment may have contributed to their current chromosomal locations, though further studies are required to confirm this mechanism.

The remaining NBPF genes present the LINE insertion in their sequences and are closely related between each other, possibly resulting from the insertion of a LINE into one of the *PDE4DIP*-derived duplicated sequences. Phylogenetic analyses of cDNA, CDS and protein trees (Figure S3; Figure 7) show that *NBPF26’s* sequences and transcripts are widely distributed across the branches of those trees, suggesting that *NBPF26* may represent the closest sequence to the original *PDE4DIP* segment containing the LINE insertion, consistent with the findings of Fiddes et al., which identify *NBPF26* as the most closely related to ancestral *NBPF* genes [9]. For clarity, we denominated the *PDE4DIP*-derived sequence with the LINE insertion as *preNBPF26*. We propose that *PDE4DIP* had multiple segmental duplications from which emerged the ancestral LINE-less group and *preNBPF26*, from which emerged the rest of the NBPF members, including its modern counterpart, *NBPF26*. Certain gene clusters warrant special attention due to their phylogenetic consistency; the cluster composed by *NBPF8*, *NBPF9*, *NBPF15*, *NBPF11* and *NBPF12* is largely conserved across different trees, being the only difference found in the protein tree (Figure 7), in which *NBPF11* is more closely related to *NBPF1* and *NBPF26*. Functionally, this cluster is notable because it includes nearly all NBPF members associated with bone growth disorders, except for *NBPF1* [47]. Another consistently grouped cluster comprises *NBPF2P*, *NBPF3* and *NBPF17P*, which always appear grouped together in the genomic DNA phylogenetic trees (Figure 5 and Figure 6) and as a brother group to the one composed of *NBPF8*, *NBPF9*, *NBPF15*, *NBPF11* and *NBPF12*. Finally, *NBPF* genes associated with *NOTCH2NL* (*NBPF10*, *NBPF14*, *NBPF19*, and *NBPF26*) show tight phylogenetic relationships with each other and with *NBPF25P*, likely reflecting a segmental microduplication in the highly unstable 1q21 region.

The CNVs, microduplications and microdeletions of the 1q21 region, have been linked to the pathological development of multiple diseases [4,5,6,17,20,22,40]. However, not all NBPF-related pathologies are explained solely by structural variations. Multiple studies have proposed that one or more NBPF genes must be altered to participate in or develop a disease. The family is commonly associated with diseases related to development and cell proliferation, including neurological pathologies such as inherited autism, schizophrenia, NIID and anomalous neuron development [4,5,6,17,18,19,20], oncological pathologies like HAL, LUSC, MPLC-LNM, PDAC, mixed ductal carcinoma, thymoma, ADC, GBM, SCC, hepatocellular carcinoma, triple-negative breast cancer, non-Hodgkin B-cell lymphoma, CRC and neuroblastoma [23,24,25,26,27,28,29,30,31,32,33,34,35,36,37,38,39,40,41,42,43,44,45,46], and bone growth disorders such as hypertelorism and macrognatism [19,47,48]. But this family has also been described in other pathological pathways, appearing in diseases like diabetes [49,50], Brugada syndrome [1], PSIS [2,3], TAPVC [52] and Mayer–Rokitansky–Küster–Hauser syndrome [53].

CNVs of NBPF members in region 1q21 have implications beyond the development of diseases, suggesting that the *NBPF* genes located in the region are under continuous processes of microduplication and microdeletion in the human population. The most studied association is the CNV-mediated balance between inherited autism/macrocephaly and schizophrenia/microcephaly. Multiple studies [4,5,6,17,22] have shown that patients with inherited autism and macrocephaly have a significantly higher number of CON1 Olduvai domain copies than controls, while patients with schizophrenia and microcephaly have significantly fewer copies. These effects suggest that the total number of copies of the CON1 subdomain may alter neuron development, particularly the total number of neurons (resulting in macro- and microcephaly) and neuronal maturing speed. Even with supporting evidence [4,5,6,17,22], the precise mechanisms underlying autism or schizophrenia remain unclear, highlighting the need to understand disease-specific signalling cascades, protein interactions, and multifactorial contributions.

These authors [4,5,6,17,22] propose a dosage-dependent genomic trade-off of the CON1 subdomain, in which a higher number of copies generate more functional neurons but potentially reduces the quality of neural connections between them. This also supports the theory of a cognitive disease continuum between autism and schizophrenia, with each condition representing opposite extremes of Olduvai domain dosage and disease severity. In this genomic trade-off, *NBPF1* plays a particularly important role due to the high variability observed of the CON1 subdomain copy number, ranging from 8.5 to 28.5 copies [4], possibly being one of the most important members involved in the regulation of this genomic trade-off. Further studies are required to validate the dosage-dependent trade-off hypothesis, the cognitive continuum model, and the broader implications of the NBPF family in the development of these conditions.

The most relevant function of the NBPF family is cell proliferation. All diseases involving NBPF genes include oncological processes or developmental malformation, depending on the nature and timing of the genetic alteration. Early or inherited alterations can produce congenital malformations [4,5,6,17,18,19,20,21,22,47,48,49,50,51,52,53], while acquired or somatic alterations may contribute to oncogenesis [23,24,25,26,27,28,29,30,31,32,33,34,35,36,37,38,39,40,41,42,43,44,45,46]. Although the specific biological functions and mechanisms of individual NBPF genes remain incompletely understood, the findings of this study strongly suggest that NBPF members represent promising targets for future research, particularly in fields related to neurodevelopment, oncogenesis, and structural genomic variation.

## 5. Conclusions

This study provides new associations between members through the construction of the first phylogenetic trees that simultaneously include all NBPF members at multiple levels (cDNA, genomic DNA, CDS and proteins). It also offers a detailed description of the diseases associated with each NBPF gene and the potential pathological mechanisms involved.

NBPF genes have an extremely complex evolution, being almost exclusive to mammals and particularly expanded in primates. Lemurs like the bushbaby (*Otolemur garnettii*) have only one copy of an NBPF gene, and humans (*Homo sapiens*) have 15 different described NBPF genes, showing the tendency of the accumulation of NBPF genes in primates.

These genes are believed to add complexity to brain development processes in primates. Glunčić et al. [54,55] propose that the most important part of this family is not the number of genes, but rather the total number of Olduvai domain copies. Humans have 302 copies, chimpanzees 138, great apes between 38 and 97, monkeys between 48 and 75 and non-primate mammals between 1 and 8 copies.

It is also worth noting the variation in the number of NBPF genes among species. The crab-eating macaque (*Macaca fascicularis*) is the primate with the highest number of *NBPF* genes with 16, followed by humans with 15 and the rabbit (*Oryctolagus cuniculus*) with 14. These differences suggest species-specific functional diversification, although cell proliferation remains a shared functional theme.

In primates, the earliest expansion of the NBPF family is found in Simiiformes (non-lemurian simians), with the most notable expansion arising in Catarrhini (apes and old-world monkeys). Within this parvorder, the superfamilies of Cercopithecidae and Hominoidea seem to possess similar numbers of NBPF genes, reinforcing the idea that this family of genes may be particularly involved in cell proliferation and their brain development functions are specific to some members.

Based on the diseases linked to NBPF gene alterations, it may be inferred that the overall family function could revolve around cell cycle regulation, with some members possibly participating in bone formation and neuronal development.

Since these genes are described in cell proliferation processes, future studies of the NBPFs involved in oncogenesis should examine the structural and regulatory state of each gene, including epigenetic alterations, CNVs, point mutations, translocations and gene fusions. Also, it is necessary to identify the location of the proteins of the NBPF genes and their specific function in relation to the cell cycle. Additionally, identifying the subcellular localization and functional roles of NBPF proteins in the cell cycle is essential. For this purpose, patient-derived biopsies or cell cultures could be studied by fluorescence microscopy using gene-specific or domain-specific antibodies, complemented with RNA-seq analyses. Flow cytometry-based isolation of cells at defined cell cycle stages would enable the precise characterization of NBPF expression and localization dynamics.

To study NBPF genes implicated in bone malformations, conventional polymerase chain reaction (PCR) and subsequent sequencing could provide an effective first approach to detect sequence variants. When paired with quantitative PCR, these analyses could reveal epigenetic alterations or the transcriptional impact of point mutations.

Finally, the role of NBPF genes in the process of brain development can be studied via targeted overexpression experiments in model animals like non-human primates and mice using CRISPR-Cas9/CRISPR-Cas9 domain editing to insert the desired genes or domains in the developing organoid or embryos. These techniques have already been used successfully in the study of human neurodevelopmental gene families [56], quantifying the effects of the presence of the genes and contrasting them with their absence (control model animal brains). For instance, *NBPF14* has been functionally characterized in embryonic mouse neocortex and chimpanzee cerebral organoids, demonstrating its role in the delamination of cortical stem and progenitor cells and the promotion of apical progenitors toward basal glial fates, requiring the co-expression of *NOTCH2NLB* to maintain apical progenitor self-renewal, as shown in the study of Eşiyok et al. [57].

Future studies could analyze the effect of the co-expression of NBPF genes with *NOTHC2NL* associated genes (*NBPF10*, *NBPF19* and *NBP26*) and other NBPF genes related to brain development like *NBPF1*.

The NBPF family is still not fully understood, but new hypotheses regarding its origins and evolution may help explain multiple complex pathologies, opening new research avenues that could significantly improve patient outcomes.

## Figures and Tables

**Table 1:** NBPF genes extracted from Ensembl (current release 113—October 2024) with its corresponding Ensembl code, location in the chromosome and the chromosome band where they are located.

Gene	Ensembl Code	Location	Chromosomal Band
*NBPF1*	ENSG00000219481	1: 16,562,319–16,613,562	1p36.13
*NBPF2P*	ENSG00000227001	1: 21,424,625–21,427,967	1p36.12
*NBPF3*	ENSG00000142794	1: 21,440,128–21,485,005	1p36.12
*NBPF4*	ENSG00000196427	1: 108,222,464–108,244,081	1p13.3
*NBPF5P*	ENSG00000243967	1: 108,376,119–108,385,897	1p13.3
*NBPF6*	ENSG00000186086	1: 108,450,282–108,471,920	1p13.3
*NBPF7P*	ENSG00000215864	1: 119,834,870–119,844,514	1p12
*NBPF8*	ENSG00000270231	1: 120,415,035–120,469,676	1p11.2
*NBPF9*	ENSG00000269713	1: 149,052,186–149,103,561	1q21.2
*NBPF10*	ENSG00000271425	1: 146,064,711–146,229,000	1q21.1
*NBPF11*	ENSG00000263956	1: 148,102,047–148,152,322	1q21.2
*NBPF12*	ENSG00000268043	1: 146,938,324–146,996,202	1q21.1
*NBPF13P*	ENSG00000283342	1: 147,099,482–147,124,285	1q21.1
*NBPF14*	ENSG00000270629	1: 148,531,385–148,679,742	1q21.2
*NBPF15*	ENSG00000266338	1: 144,421,390–144,461,676	1q21.1
*NBPF17P*	ENSG00000179571	1: 143,595,216–143,635,641	1q21.1
*NBPF18P*	ENSG00000240667	1: 152,018,662–152,022,508	1q21.3
*NBPF19*	ENSG00000271383	1: 149,475,045–149,556,361	1q21.2
*NBPF20*	ENSG00000162825	1: 145,289,900–145,425,603	1q21.1
*NBPF21P*	ENSG00000231382	3: 36,616,006–36,637,457	3p22.2
*NBPF22P*	ENSG00000205449	5: 86,282,766–86,296,309	5q14.3
*NBPF25P*	ENSG00000291005	1: 145,572,345–145,608,551	1q21.1
*NBPF26*	ENSG00000273136	1: 120,723,945–120,842,229	1p11.2
*NBPF3* (*Pan troglodytes*)	ENSPTRG00000000300	1: 20,531,551–20,575,768	-
ENSPTRG00000001028 (*Pan troglodytes*)	ENSPTRG00000001028	1: 108,883,957–108,908,573	-
*PDE4DIP*	ENSG00000178104	1: 148,808,140–149,048,286	1q21.2

## Data Availability

Data will be made available on request.

## Supplementary Materials

The following supporting information can be downloaded at: https://www.mdpi.com/article/10.3390/jdb14010010/s1. Figure S1: Genomic DNA phylogenetic tree obtained through neighbour joining methods with its bootstrap results in each branch (*NBPF2P* was excluded from this tree because an alignment with it has no gap-free spaces, which are needed for the neighbour joining methods). Figure S2: Genomic DNA + *NOTCH2NL* genomic DNA phylogenetic tree obtained through neighbour joining methods with its bootstrap results in each branch (*NBPF2P* was excluded from this tree because an alignment with it has no gap-free spaces, which are needed for the neighbour joining methods). Figure S3: cDNA phylogenetic trees obtained through UPGMA methods (not compatible with bootstrap analysis). Figure S4: CDS phylogenetic trees obtained through UPGMA methods (not compatible with bootstrap analysis). Table S1: All articles systematically reviewed ordered by bibliographic reference, DOI link, related pathological group, platform of extraction and NBPF genes associated.

## Abbreviations

The following abbreviations are used in this manuscript:

*NBPF*	Neuroblastoma breakpoint family
HLS	Human lineage specific
PSIS	Pituitary stalk interruption syndrome
CNVs	Copy number variations
SDS	Social diagnostic score
CDS	Communication diagnostic score
NIID	Neuronal intranuclear inclusion disease
HAL	Hepatoid adenocarcinoma of the lung
LUSC	Lung squamous cell carcinoma
MPLC	Multiple primary lung cancer
LNM	Lymph node metastasis
AML	Acute myeloid leukemia
PDAC	Pancreatic ductal adenocarcinoma
TMB	Tumour mutational burden
SCC	Small-cell carcinoma
ADC	Adenocarcinoma
GBM	Glioblastoma multiforme
IERs	Intragenic exon rearrangements
CRC	Colorectal cancer
ACC	Adrenocortical carcinoma
LUAD	Lung adenocarcinoma
BRCA	Breast-invasive carcinoma
T2DM	Type 2 diabetes mellitus
T1DM	Type 1 diabetes mellitus
WES	Whole-exome sequencing
TAPVC	Total anomalous pulmonary venous connection
LINE	Long interspersed nuclear element
PCR	Polymerase chain reaction
