# Supplementary material for: Evolutionary Restructuring and Systematic Review of the NBPF Gene Family: Comparative Genomics, Functional Divergence, and Disease-Linked Pathways
Source: J Dev Biol. 2026 Feb 24;14(1):10. doi: 10.3390/jdb14010010 (PMC13028154; doi:10.3390/jdb14010010)
Supplement: Supplementary file 1 [file jdb-14-00010-s001.zip › jdb-4028461-supplementary.pdf]

Table S1: all articles systematically reviewed ordered by bibliographic reference, DOI link, related pathological group, platform of extraction and NBPF genes associated.

Reference	DOI	Pathological group	Platform	NBPFs associated
1	https://doi.org/10.5808/gi.22047	Other	PubMed	*NBPF1, NBPF11 and NBPF12*
2	https://pubmed.ncbi.nlm.nih.gov/32304364	Other	PubMed	*NBPF10*
3	https://doi.org/10.1155/2020/5401738	Other	PubMed	*NBPF9*
4	https://doi.org/10.1176/appi.ajp.2018.18080993	Neurological	PubMed	*NBPF1 and NBPF14*
5	https://doi.org/10.1093/bfgp/elz016	Neurological	ScienceDirect	*NBPF1*
6	https://doi.org/10.1038/tp.2015.192	Neurological	PubMed	Olduvai domains
7	https://doi.org/10.1007/s12104-022-10068-5	Neurological	PubMed	Olduvai domains
8	https://doi.org/10.1016/j.ijbiomac.2022.12.260	Neurological	PubMed	Olduvai domains
9	https://doi.org/10.1007/s00439-019-02018-4	Neurological	PubMed	*NBPF10, NBPF14, NBPF19 and NBPF26*
10	https://doi.org/10.1093/molbev/msi222	Oncological	PubMed	*NBPF1*
11	https://doi.org/10.1016/j.biocel.2013.07.022	Oncological	ScienceDirect	*NBPF1 and NBPF12*
17	https://doi.org/10.1101/2024.10.21.619278	Neurological	PubMed	*NBPF1*
18	https://doi.org/10.12688/f1000research.14451.3	Neurological	PubMed	*NBPF14*
19	https://doi.org/10.3389/fgene.2021.728816	Neurological and bone growth	PubMed	*NBPF15*
20	https://doi.org/10.1038/s41588-019-0458-z	Neurological	PubMed	*NBPF19*
21	https://doi.org/10.3390/diagnostics15121542	Neurological	PubMed	Olduvai domains
22	https://pmc.ncbi.nlm.nih.gov/articles/PMC11075447/	Neurological	PubMed	Olduvai domains
23	https://doi.org/10.3389/fphar.2022.945038	Oncological	PubMed	*NBPF26*
24	https://doi.org/10.1007/s12094-021-02638-1	Oncological	PubMed	*NBPF26*
25	https://doi.org/10.1038/s41417-022-00572-0	Oncological	PubMed	*NBPF20*
26	https://doi.org/10.3390/biomedicines9060618	Oncological	PubMed	*NBPF20*
27	https://doi.org/10.3389/fgene.2021.763636	Oncological	PubMed	*NBPF15*
29	https://doi.org/10.1038/s41598-019-38878-z	Oncological	PubMed	*NBPF14*
30	https://doi.org/10.1038/leu.2016.267	Oncological	PubMed	*NBPF14*
31	https://doi.org/10.1016/j.gene.2014.06.042	Oncological	ScienceDirect	*NBPF10*
32	https://doi.org/10.1007/s10555-015-9558-0	Oncological	PubMed	*NBPF1, NBPF9 and NBPF10*
33	https://doi.org/10.3389/fmolb.2021.800679	Oncological	PubMed	*NBPF1 and NBPF10*
34	https://doi.org/10.1007/s10552-022-01574-x	Oncological	PubMed	*NBPF12*
35	https://doi.org/10.1186/s40246-023-00511-6	Oncological	PubMed	*NBPF1 and NBPF10*
36	https://doi.org/10.1007/s12185-019-02766-z	Oncological	PubMed	*NBPF8*
37	https://doi.org/10.18632/oncotarget.19480	Oncological	PubMed	*NBPF7*
38	https://doi.org/10.1016/j.yexcr.2010.01.019	Oncological	ScienceDirect	*NBPF1*
39	https://doi.org/10.1371/journal.pone.0002207	Oncological	PubMed	*NBPF1*
40	https://doi.org/10.1186/s12885-015-1408-5	Oncological	PubMed	*NBPF1*
41	https://doi.org/10.1016/j.heliyon.2024.e34535	Oncological	ScienceDirect	*NBPF1*

42	https://doi.org/10.1186/s40246-022-00424-w	Oncological	ScienceDirect	*NBPF1*
43	https://doi.org/10.1016/j.ygeno.2020.06.038	Oncological	ScienceDirect	*NBPF1 and NBPF10*
44	https://doi.org/10.1186/s13148-025-01906-z	Oncological	PubMed	*NBPF1*
45	https://doi.org/10.3389/fendo.2022.950326	Oncological	PubMed	*NBPF1*
46	https://doi.org/10.7150/jca.37017	Oncological	PubMed	*NBPF1, NBPF4, NBPF11, NBPF12, NBPF14, NBPF16, NBPF20 and PDE4DIP*
47	https://doi.org/10.21203/rs.2.18399/v1	Bone growth	Google Scholar	*NBPF1 and NBPF15*
48	https://doi.org/10.1016/j.ajodo.2018.08.020	Bone growth	ScienceDirect	*NBPF8 and NBPF9*
59	https://doi.org/10.3390/genes14122207	Other	Google Scholar	*NBPF20*
50	https://doi.org/10.1210/clinem/dgab488	Other	PubMed	*NBPF20*
51	https://doi.org/10.1507/endocrj.ej22-0178	Other	PubMed	*NBPF1*
52	https://doi.org/10.3389/fgene.2018.00559	Other	PubMed	*NBPF3*
53	https://doi.org/10.1016/j.eurox.2019.100089	Other	ScienceDirect	*NBPF10*
54	https://doi.org/10.1038/s41598-023-41517-3	Neurological	PubMed	Olduvai domains
55	https://doi.org/10.26508/lsa.202101306	Neurological	PubMed	Olduvai domains
56	https://doi.org/10.1016/j.cell.2024.08.052	Neurological	PubMed	*NBPF10 and NBPF12*
57	https://doi.org/10.1126/sciadv.ads7543	Neurological	ScienceDirect	*NBPF14*

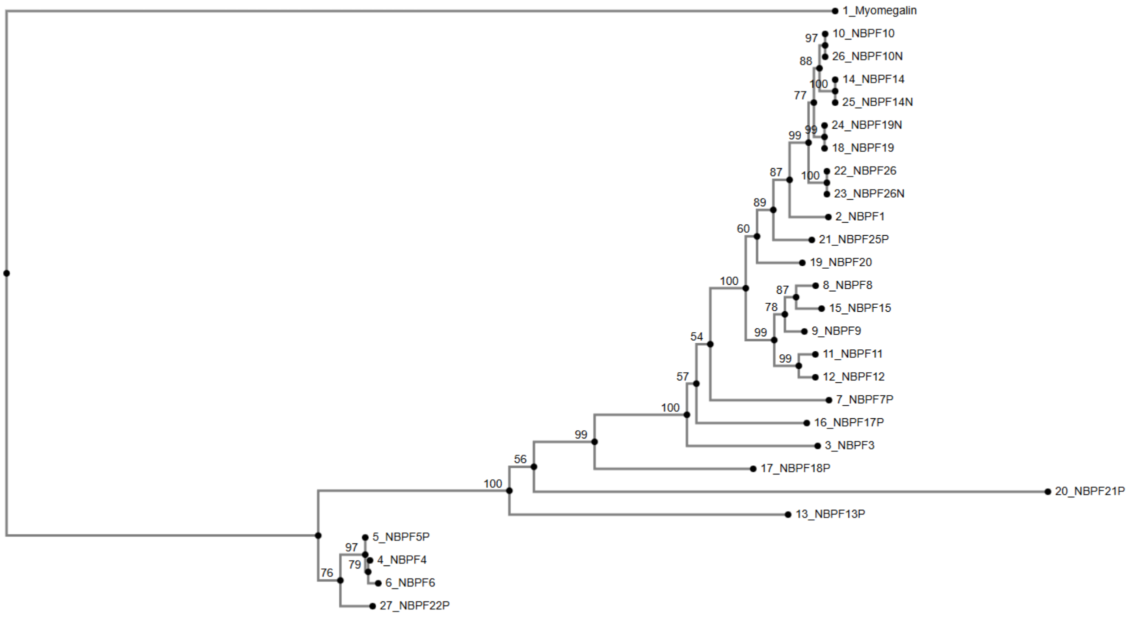

Figure S1: Genomic DNA phylogenetic tree obtained through Neighbour joining methods with its bootstrap results in each branch (*NBPF2P* was excluded from this tree because an alignment with it has no gap-free spaces, which are needed for the Neighbour joining methods)

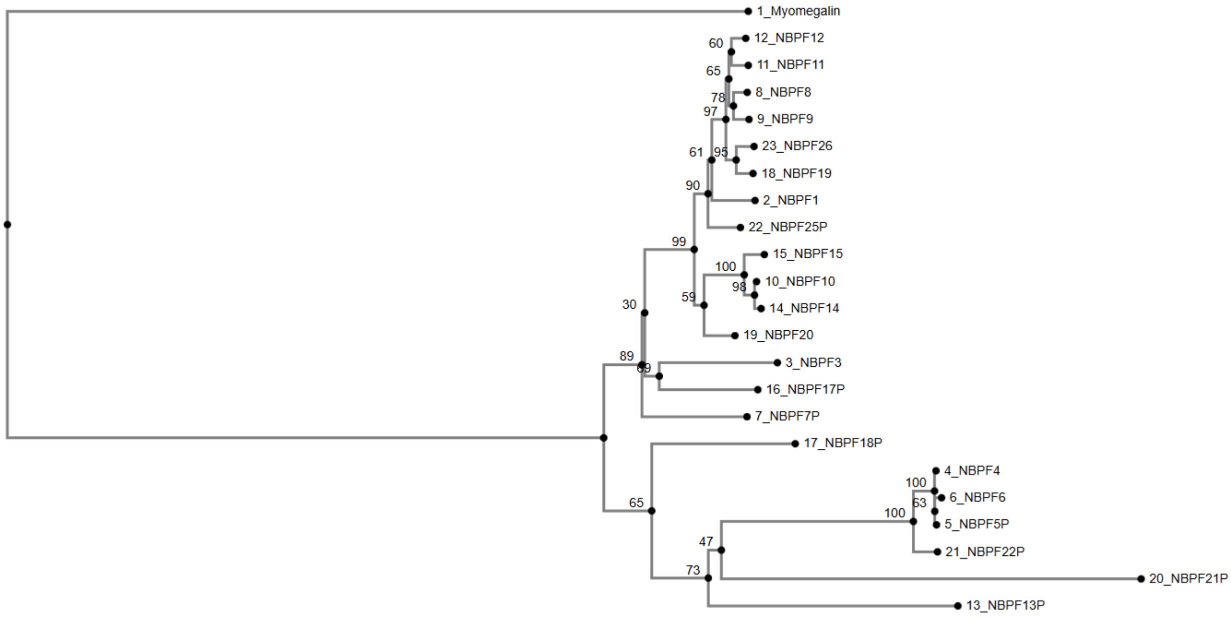

Figure S2: Genomic DNA + NOTCH2NL genomic DNA phylogenetic tree obtained through Neighbour joining methods with its bootstrap results in each branch (NBPF2P was excluded from this tree because an alignment with it has no gap-free spaces, which are needed for the Neighbour joining methods)

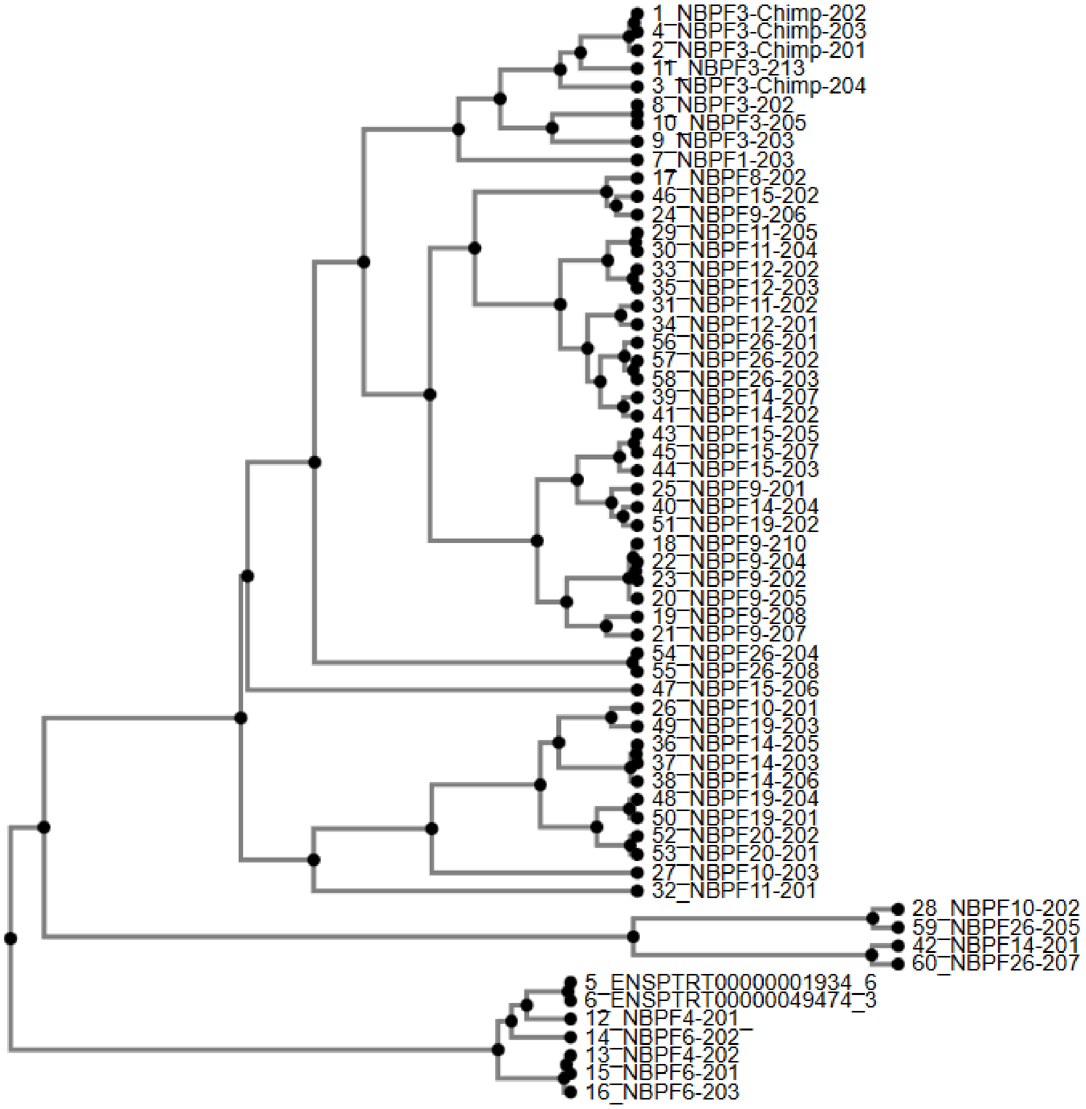

Figure S3: cDNA phylogenetic tree obtained through obtained through UPGMA methods (not compatible with bootstrap analysis).

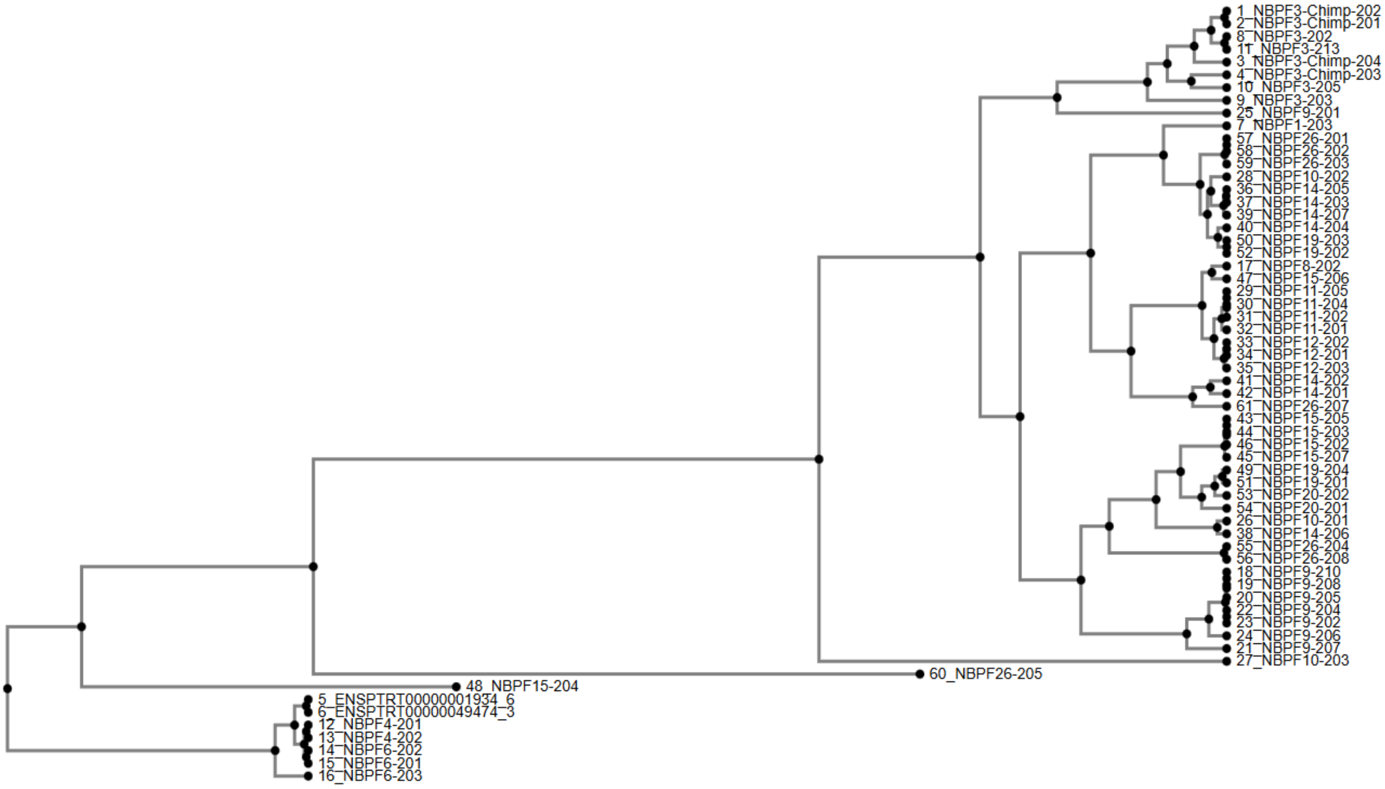

Figure S4: CDS phylogenetic trees obtained through UPGMA methods (not compatible with bootstrap analysis).
